# Intra-Atrial Conduction Delay Revealed by Multisite Incremental Atrial Pacing is an Independent Marker of Remodeling in Human Atrial Fibrillation

**DOI:** 10.1016/j.jacep.2017.02.012

**Published:** 2017-09

**Authors:** Steven E. Williams, Nick W.F. Linton, James Harrison, Henry Chubb, John Whitaker, Jaswinder Gill, Christopher A. Rinaldi, Reza Razavi, Steven Niederer, Matthew Wright, Mark O'Neill

**Affiliations:** a Division of Imaging Sciences and Biomedical Imaging, King’s College London, London, United Kingdom; b Cardiovascular Division, Guy’s and St. Thomas’ NHS Foundation Trust, London, United Kingdom

**Keywords:** AF substrate, atrial fibrillation, atrial remodeling, electrophysiology testing, ADT, activation dispersion time, AF, atrial fibrillation, CS, coronary sinus, ΔED, rate dependence of electrogram duration, ED, electrogram duration, ERP, effective refractory period, ΔEV, rate dependence of electrogram voltage, EV, electrogram voltage, HRA, high right atrium, LA, left atrial, PAF, paroxysmal AF, S1S2block, the shortest S1S2 coupling interval that conducts from pacing site to left atrium, S1S2delay, the shortest S1S2 coupling interval conducting without decrement to the left atrium

## Abstract

**Objectives:**

This study sought to characterize direction-dependent and coupling interval–dependent changes in left atrial conduction and electrogram morphology in uniformly classified patients with paroxysmal atrial fibrillation (AF) and normal bipolar voltage mapping.

**Background:**

Although AF classifications are based on arrhythmia duration, the clinical course, and treatment response vary between patients within these groups. Electrophysiological mechanisms responsible for this variability are incompletely described.

**Methods:**

Intracardiac contact mapping during incremental atrial pacing was used to characterize atrial conduction, activation dispersion, and electrogram morphology in 15 consecutive paroxysmal AF patients undergoing first-time pulmonary vein isolation. Outcome measures were vulnerability to AF induction at electrophysiology study and 2-year follow-up for arrhythmia recurrence.

**Results:**

Conduction delay showed a bimodal distribution, occurring at either long (high right atrium pacing: 326 ± 13 ms; coronary sinus pacing: 319 ± 16 ms) or short (high right atrium pacing: 275 ± 11 ms; coronary sinus pacing: 271 ± 11 ms) extrastimulus coupling intervals. Arrhythmia recurrence was found only in patients with conduction delay at long extrastimulus coupling intervals, and patients with inducible AF were characterized by increased activation dispersion (activation dispersion time: 168 ± 29 ms vs. 136 ± 11 ms). Electrogram voltage and duration varied throughout the left atrium, between patients, and with pacing site but were not correlated with AF vulnerability or arrhythmia recurrence.

**Conclusions:**

Within the single clinical entity of paroxysmal AF, incremental atrial pacing identified a spectrum of activation patterns correlating with AF vulnerability and arrhythmia recurrence. In contrast, electrogram morphology (characterized by electrogram voltage and duration) was highly variable and not associated with AF vulnerability or recurrence. An improved understanding of the electrical phenotype in AF could lead to improved mechanistic classifications.

## Graphical abstract

**Figure:**
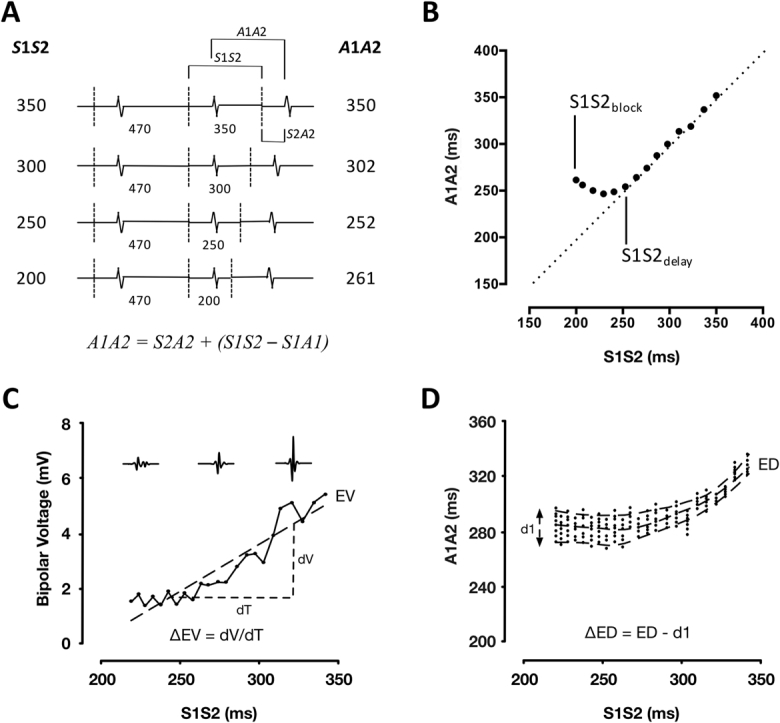

Atrial fibrillation (AF) classifications that are used to recommend treatment decisions are based on AF episode duration (1); however, a number of observations suggest that within classification categories, AF is a structurally and electrically diverse arrhythmia. First, ablation shows variable success between apparently similar patients when controlling for comorbidities and left atrial (LA) dimensions. Second, some patients require multiple ablation procedures to achieve freedom from AF despite durable pulmonary vein isolation, whereas others require only a single procedure. Finally, the natural history of AF varies, with differing rates of progression to persistent AF seen. In line with these observations, reduced conduction velocity and shortened refractoriness have been associated with increasing severity, duration or recurrence of AF in some (2), but not all 3, 4, studies.

Recently, structural (5) and voltage-defined (6) targets for intervention beyond pulmonary vein isolation have been proposed. Although low voltage is correlated with the presence of magnetic resonance imaging indices of atrial fibrosis (7), preservation of normal bipolar voltage does not imply absence of fibrosis (8). Therefore, bipolar voltage measured only during sinus rhythm or fixed coupling interval pacing may fail to detect atrial structural change. Furthermore, whether LA conduction abnormalities or morphological electrogram changes occur in apparently healthy atria (with normal bipolar voltage) is unknown.

We hypothesized that direction- and coupling interval–dependent changes in electrogram morphology and timing in patients with apparently healthy atria may differentiate between truly normal atria and those with underlying substrate change. In this study, incremental atrial pacing was used to measure electrogram voltage (EV), duration, conduction, and activation dispersion in a group of uniformly classified patients with paroxysmal AF (PAF) and otherwise normal atria.

## Methods

### Patient selection and clinical procedures

Ethical approval was granted by the National Research Ethics Service (10/H0802/77), and all participants gave written informed consent for study inclusion. The research conformed to the principles described in the Declaration of Helsinki. Patients with ischemic heart disease, cardiac surgery, or structural heart disease were excluded. Antiarrhythmic drugs, including calcium channel blockers, were stopped at least 5 half-lives before ablation. Amiodarone was stopped at least 6 weeks before ablation. All clinical procedures were performed under general anesthesia. Following femoral access and trans-septal puncture, 2 8.5-F SR0 long sheaths and a PentaRay mapping catheter (Biosense Webster, Diamond Bar, California, 4-4-4 mm electrode spacing) were advanced into the left atrium. Decapolar (St. Jude Medical, St. Paul, Minnesota) and pentapolar (Bard Electrophysiology, Natick, Massachusetts) catheters were positioned in the coronary sinus (CS) and high right atrium (HRA), respectively.

### Pacing protocol

**Figure 1:**
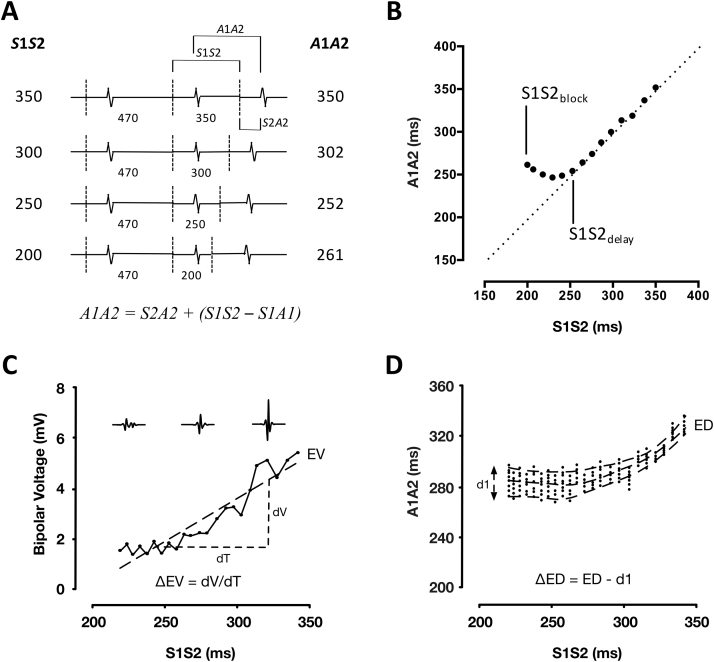
Study Protocol **(A)** Pacing protocol with measured intervals marked. **(B)** Schematic representation of A1A2 times as a function of extrastimulus coupling interval illustrating measurement of S1S2_delay_ and S1S2_block_ times. Relationship between electrogram voltage **(C)** or electrogram duration **(D)** and extrastimulus coupling interval with study parameters marked. ΔED = rate dependence of electrogram duration; ED = electrogram duration; EV = electrogram voltage; ΔEV = rate dependence of electrogram voltage; S1S2_block_ = shortest S1S2 conducted to the left atrial recording site; S1S2_delay_ = shortest S1S2 interval that conducted without decrement to the left atrium.

The pacing protocol was delivered using a custom-built, institutionally approved stimulator (Online Figures 1 and 2). The protocol was designed to allow LA response to a full range of extrastimulus coupling intervals (down to atrial effective refractory period [ERP]) to be recorded at more than 100 sites per chamber. As such, the drive train was reduced to 2 beats (470 ms) followed by a single premature extrastimulus. The S1S2 coupling interval was reduced continuously without operator interference in 2% steps from 350 ms to 200 ms or loss of capture (e.g., 470-470-350, 470-470-343) (Figure 1A). All pacing stimuli were delivered at a voltage of at least twice threshold, with a pulse width of 2 ms. The PentaRay catheter was sequentially maneuvered to multiple sites in the body of the left atrium, and bipolar electrograms were recorded throughout in response to complete S1S1S2 pacing trains delivered from the HRA and mid-CS.

### Signal processing

Electrograms were digitized using LabSystem Pro-EP (Bard Electrophysiology) at 16-bit/4 kHz. Signal processing was performed offline (MATLAB 8.2, MathWorks, Natick, Massachusetts). Pacing timing was determined from the paced channel (HRA or CS). The first pacing cycle was used to determine the noise threshold and discarded from subsequent analysis. A2 electrograms were rejected from analysis if the signal was far-field, there was fusion with an atrial ectopic beat, or the 2 preceding S1 beats failed to capture the left atrium. A band-pass filter (30 to 500 Hz) was applied to bipolar recordings, and the noise threshold defined as signal mean ± 3 SD of the 100 ms preceding the S2 component of the first cycle.

### Electrogram analysis

Electrogram analysis was performed to quantify electrogram morphology, conduction response, activation dispersion, and atrial refractoriness. Criteria used are summarized in Table 1.

**Table 1:** Description of Study Parameters

	Metric	Description	Unit
Electrogram morphology	EV	Peak-to-peak bipolar voltage at longest extrastimulus coupling interval	mV
	ΔEV	Change in EV per unit change in extrastimulus coupling interval	mV/s
	ED	Time interval between first and last sharp electrogram components at longest extrastimulus coupling interval	ms
	ΔED	Maximal increase in ED at short extrastimulus coupling interval compared to baseline ED	ms
Conduction	S1S2_delay_	The shortest S1S2 coupling interval conducting to the left atrium with S1S2 = A1A2	ms
	S1S2_block_	The shortest S1S2 coupling interval that conducts from pacing site to left atrium	ms
	ADT	Longest LA activation time (measured at shortest S1S2 coupling interval) minus shortest LA activation time (measured at longest S1S2 coupling interval)	ms
Refractoriness	ERP	The longest S1S2 coupling interval at which S2 failed to produce local capture	ms

ADT = activation dispersion time; ΔED = rate dependence of electrogram duration; ED = electrogram duration; ERP = effective refractory period; ΔEV = rate dependence of electrogram voltage; EV = electrogram voltage; LA = left atrial.

#### Electrogram morphology

Electrogram peak-to-peak bipolar voltage (EV) and duration (ED) were determined from the longest S2 pacing cycle length analyzed at each site. The rate dependence of electrogram amplitude was quantified by calculating the gradient of the best-fit line through S1S2 versus amplitude curves. This parameter (rate dependence of electrogram voltage [ΔEV], mV/s) represents the change in EV with coupling interval (Figure 1C). The rate dependence of electrogram duration was quantified from S1S2-A1A2 plots for each recording site. For each S2 cycle length, the 5th and 95th centile of A2 electrogram components were taken as the beginning and end of local activation, respectively. Change in electrogram duration was quantified as the longest duration between these curves minus the duration at baseline (ΔED, ms), which represents the maximal change in electrogram duration with coupling interval (Figure 1D). Electrogram fractionation was quantified by counting the number of peaks/troughs greater than the noise threshold for each filtered electrogram.

#### Conduction responses

To assess the impact of coupling interval on electrogram timing, S1S2-A1A2 curves were plotted. These curves were characterized by: 1) an A1A2 equal to S1S2 at long pacing cycle lengths; 2) a minimum achievable A1A2 at short S1S2; and 3) a curved transition period between these regions. A hyperbola with asymptotes at y = c and y = x was fitted to these data. S1S2_block_ was defined as the shortest S1S2 conducted to the LA recording site. The shortest S1S2 interval that conducted without decrement to the left atrium (S1S2_delay_) was determined as the transition point of the curve (where A1A2 becomes greater than S1S2). S1S2_delay_ represents the coupling interval at which initial delay of the A2 electrogram occurs (Figure 1B).

#### Activation dispersion

LA activation dispersion was assessed from the appearance of conduction curves representing all recording sites for an individual atrium. Activation dispersion was quantified by analyzing conduction parameters for regions close to and distant from the pacing site. Geodesic distances across the surface of the LA electroanatomic shell were calculated from the site of earliest activation to the recording site in the left atrium (Online Figure 3). Activation dispersion time (ADT) (ms) was defined as the difference between the latest and earliest activation times within an LA segment during extrastimulus testing. ADT was calculated for all points with geodesic distances greater and <60 mm (the median of overall path length distributions).

#### Atrial refractoriness

Atrial ERP was measured at 2 sites (HRA and mid-CS) at a single basic cycle length (470 ms). ERP was defined as the longest S1S2 extrastimulus coupling interval that failed to produce local atrial capture.

### AF vulnerability and follow-up

AF vulnerability was assessed during pacing from HRA and CS. Sustained AF was defined as AF triggered by the pacing protocol and lasting for more than 30 s (9). Where AF resolved to an organized tachycardia, overdrive pacing was used to terminate the tachycardia. Electrical cardioversion was performed if AF/atrial tachycardia persisted beyond 5 min. Following electrical cardioversion, no further measurements were taken within a blanking period of 10 min (4). Time to arrhythmia recurrence was recorded over 2 years of routine clinical follow-up consisting of 3 to 6 monthly clinic reviews combined with 48-h ambulatory monitoring.

### Statistical tests

Data analysis was performed using GraphPad Prism 6.0c (GraphPad Software, San Diego, California). Data are represented as mean ± SD or median (interquartile range) for categorical variables. Categorical variables were compared with Fisher exact test. Continuous variables were compared using Student 2**-**tailed *t* test. Group means of 3 or more variables were compared with analysis of variance. Comparisons of differences between patients were expressed using standardized differences. Standardized differences of 0.2, 0.5, and 0.8 are taken to represent small, medium, and large effect sizes, respectively. Kaplan-Meier curves were compared with the log-rank test. A value of p < 0.05 was considered significant.

## Results

Fifteen consecutive patients (40% male; age: 64 ± 11 years) undergoing first-time pulmonary vein isolation were studied. All patients had PAF with similar symptom duration (2.2 ± 1.7 years) and LA dimensions (4.0 ± 0.6 cm). The median CHA_2_DS_2_-VASc score was 2 (interquartile range: 0.5 to 3.5). There were no regions of LA low voltage (<0.3 mV) (10) during baseline pacing. In total, 23,142 electrograms were recorded at 120 ± 31 sites per left atrium. An operator blinded to the case details inspected the quality of every electrogram (Online Figure 4). A total of 34.6 ± 17.6% (HRA pacing) and 35.9 ± 17.7% (CS pacing) of PentaRay bipolar pairs yielded suitable electrograms for analysis.

### Electrogram morphology

**Figure 2:**
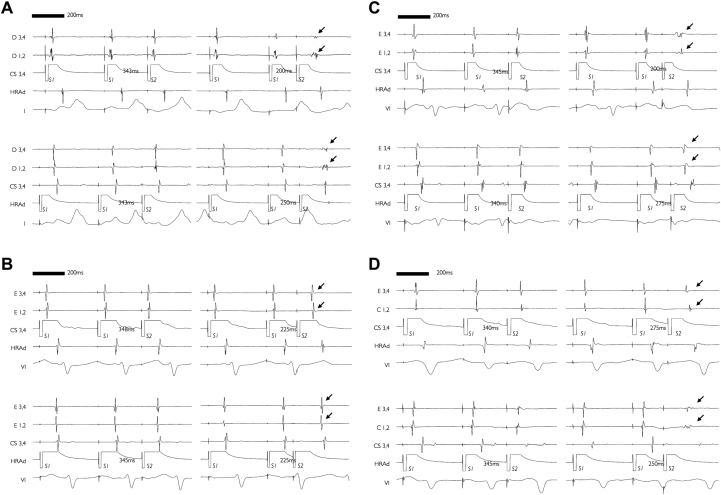
Electrogram Fractionation: Dependence on Activation Direction Recordings showing electrogram fractionation independent of, or dependent on, activation direction are shown on the **left and right panels**, respectively. **(A)** Electrograms showing fractionation of A2 components **(arrows)** during CS pacing and HRA pacing. **(B)** Absence of A2 fractionation **(arrows)** during CS pacing or HRA pacing. **(C)** Fractionation of A2 components **(arrows)** only during CS pacing. **(D)** Fractionation of A2 components **(arrows)** only during HRA pacing. CS = coronary sinus; HRA = high right atrium.

**Figure 3:**
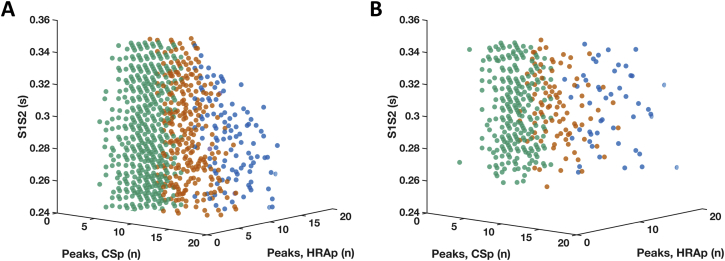
Effect of Extrastimulus Coupling Interval on Electrogram Fractionation Number of electrogram peaks during HRAp and CSp (x- and y-axes) plotted against extrastimulus coupling interval (z-axis). **(A)** Increased direction-dependent **(orange)** and direction-independent **(blue)** fractionation with shortening of S1S2 interval. **(B)** Direction-dependent **(orange)** and direction-independent **(blue)** fractionation present at both long and short S1S2 intervals. CSp = CS pacing; HRAp = high right atrial pacing.

A spectrum of extrastimulus atrial electrogram (A2) morphologies were seen. Short coupled extrastimuli resulted in low-amplitude, prolonged-duration electrograms in some cases (Figure 2A) (n = 8), whereas other cases were characterized by uniform nonfractionated electrograms even at short coupling intervals (Figure 2B) (n = 7). The occurrence of prolonged-duration, low-voltage electrograms was often dependent on pacing site, indicating an interrelationship between electrogram fractionation and activation direction. Examples of fractionation arising from CS pacing only or HRA pacing only are given in Figures 2C and 2D. Overall, this unidirectional fractionation was more common than bidirectional fractionation (13.0 ± 8.4% vs. 3.0 ± 4.0% of recording sites per atria; standardized difference: 1.44). Of the cases showing fractionated local electrograms, one-half (n = 4) showed progressive fractionation as the extrastimulus coupling interval was reduced (Figure 3).

**Figure 4:**
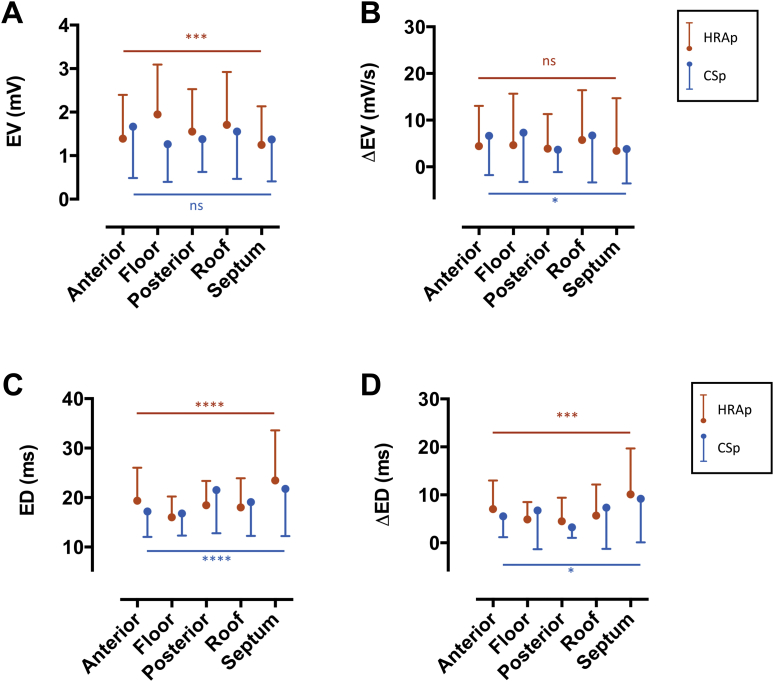
Electrogram Voltage and Duration **(A)** Regional distributions of baseline EV. **(B)** Regional distributions of electrogram voltage change with extrastimulus coupling interval (ΔEV). Significance levels are shown for differences between LA regions under HRAp **(orange)** and CSp **(blue)**. **(C)** Regional distributions of baseline ED. **(D)** Regional distributions of electrogram duration change with extra-stimulus coupling interval (ΔED). Region definitions are defined in Online Figure 5. Abbreviations as in Figures 1, 2, and 3.

Left atrial EV ranged from 0.7 to 2.7 mV for HRA pacing and 1.0 to 2.4 mV for CS pacing. Intra-atrial regional differences in voltage were detectable during HRA pacing, but not during CS pacing, with HRA pacing revealing a significant voltage gradient between anterior/septal regions (low EV) and floor/posterior/roof regions (high EV) (Figure 4A). Despite regional variations in baseline voltage, the ΔEV was uniform throughout the left atrium under HRA pacing conditions (Figure 4B). On a point-by-point basis, there was a weak but significant correlation between HRA pacing and CS pacing EV (R^2^ = 0.2, p < 0.0001), indicating that EV is partially (but not completely) independent of activation direction.

ED ranged from 11 to 57 ms for HRA pacing and from 8 to 65 ms for CS pacing. During both HRA pacing and CS pacing, ED varied between LA regions, with the longest ED recorded at the septum (Figure 4C). There was however no clear point-by-point relationship between ED recorded under HRA pacing and CS pacing conditions, confirming the interrelationship between electrogram fractionation and activation direction noted from the raw electrograms discussed previously. The ΔED was more pronounced in regions of prolonged ED during baseline pacing (Figure 4D). On a point-by-point basis, there was no significant relationship between ΔED measured under HRA pacing and CS pacing conditions.

### Atrial refractoriness

Mean HRA and CS ERPs at a basic cycle length of 470 ms were 239 ± 21 ms and 231 ± 19 ms, respectively (p = 0.4763). There was significant variability in ERP between study patients (HRA ERP range: 208 to 286 ms; CS ERP range: 200 to 274 ms).

### Conduction response

**Figure 5:**
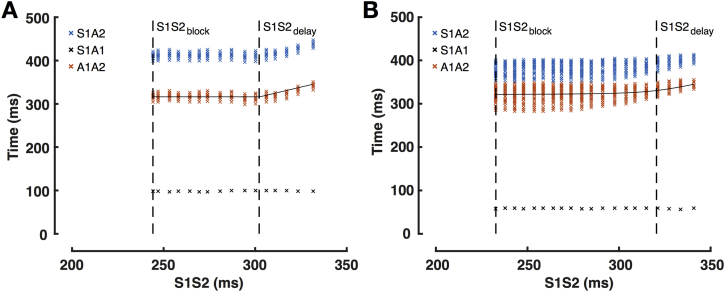
Conduction Curves Typical S1S2-A1A2 curves from 2 cases with early **(A)** and late **(B)** conduction delay. **Black crosses** show the time from the S1 stimulus preceding the extrastimulus to local left atrial activation (A1 local electrogram peak). **Blue crosses** show the timing of individual peaks of the A2 left atrial electrogram, timed to the preceding S1 extrastimulus. **Orange crosses** show individual peaks of the A2 electrogram, timed to the preceding A1 electrogram. Abbreviations as in Figure 1.

**Figure 6:**
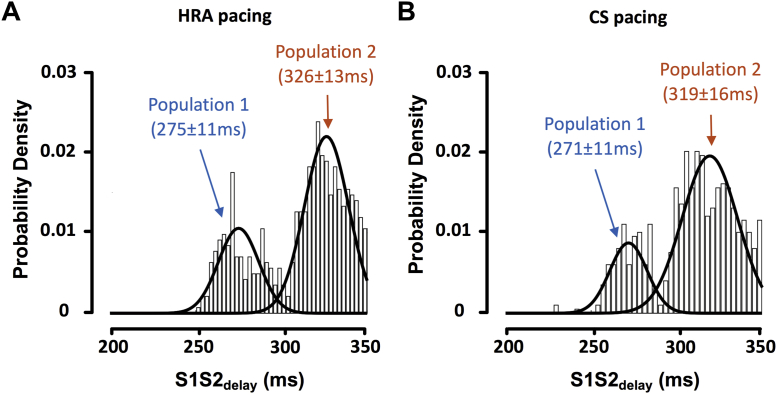
Conduction Response Frequency Distributions Overall distributions of S1S2_delay_ are shown for HRA and CS pacing. S1S2_delay_ showed a bimodal distribution represented by a Gaussian mixed model. **(A)** HRA pacing. Peaks at 275 ± 11/326 ± 13 ms (K-S p = 0.31: sample is not significantly different to the bimodal distribution). **(B)** CS pacing. Peaks at 271 ± 11/319 ± 16 ms (K-S p = 0.52: sample is not significantly different to the bimodal distribution). K-S = Kolmogorov-Smirnov; other abbreviations as in Figures 1 and 2.

A spectrum of conduction curves was seen, ranging from long conduction delay/block without electrogram prolongation/fractionation to short conduction delay/block with pronounced electrogram fractionation (Figure 5). Conduction block was quantified with S1S2_block_. S1S2_block_ was evenly distributed on the range of 200 to 300 ms for both HRA pacing and CS pacing. Conduction delay was quantified with S1S2_delay_. S1S2_delay_ showed a bimodal distribution with peaks for HRA pacing at 275 ± 11 ms and 326 ± 13 ms and for CS pacing at 271 ± 11 ms and 319 ± 16 ms (Figure 6). Within a single case, measured S1S2_delay_ was uniform across the left atrium and was independent of activation direction (S1S2_delay_ during CS pacing vs. S1S2_delay_ during HRA pacing; R^2^ = 0.6674, p < 0.0001).

### Activation dispersion

**Figure 7:**
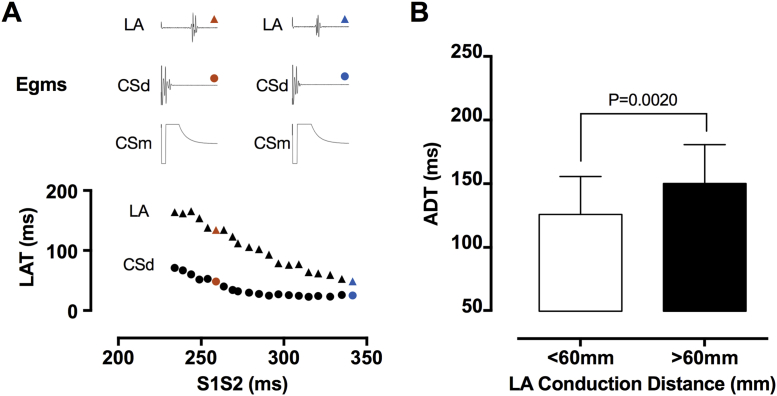
Components of Intra-Atrial Conduction Delay **(A)** Relationship between CS activation time (“pacing latency,” **circles**) and LA activation time (“conduction delay,” **triangles**). **(B)** Activation dispersion time represents the difference between earliest and latest LA activation and is illustrated for regions of the chamber <60 mm and >60 mm from the earliest LA activation site. CSd = distal coronary sinus; CSm = mid coronary sinus; LA = left atrium; LAT = local activation time.

Conduction delay close to the pacing site was correlated with S1S2_delay_ (R^2^ = 0.83, p < 0.0001), but insufficient to explain the overall conduction delay seen in the left atrium (circles vs. triangles) (Figure 7A). The LA local activation time progressively delayed as the recording site moved more distant from the stimulation site, although a number of cases showed reduced or absent intra-atrial decrement. Activation dispersion time (ADT, change in local activation time at long vs. short coupling intervals) was significantly greater for recording sites more than 60 mm from the earliest LA activation (Figure 7B). Prolonged S1S2_delay_ was correlated with increased activation dispersion (R^2^ = 0.42 for correlation between ADT >60 mm and S1S2_delay_, p = 0.0089).

### AF vulnerability and follow-up

AF was induced in 9 of 15 (60%) patients. HRA pacing induced AF more often than CS pacing (8 of 15 [53%] vs. 4 of 15 [27%], standardized difference = 0.57). In the patients in whom AF was induced, a spectrum of AF vulnerability was present with extrastimulus testing resulting in AF after 0% to 73% of HRA pacing runs and 0% to 50% of CS pacing runs (mean: 34 ± 25% for HRA pacing and 15 ± 20% for CS pacing; standardized difference = 0.87). S2 interval at AF induction was longer for HRA pacing than CS pacing (287 ± 20 ms vs. 272 ± 33 ms, standardized difference = 0.55). There were only small differences in electrogram morphology parameters between patients in whom AF was induced and those in whom AF was not induced (Table 2), but dispersion of LA activation was markedly greater (168 ± 29 ms vs. 136 ± 11 ms, standardized difference = 0.94). The S2 coupling interval at AF induction was significantly shorter than S1S2_delay_ (281 ms vs. 321 ms, p = 0.0011), indicating that S1S2_delay_ measurement is feasible without AF induction.

**Table 2:** Electrogram Morphology in Patients With and Without AF Induction

Pacing Site	Study Parameter	AF-Inducible	AF-Noninducible	Standardized Difference (d)
HRA	EV (mV)	1.4 ± 0.6	1.6 ± 0.4	0.39
	ED (ms)	29.0 ± 4.7	29.4 ± 4.9	0.07
	ΔEV (mV/s)	5.9 ± 4.5	4.5 ± 4.2	0.33
	ΔED (ms/ms)	25.4 ± 16.0	27.6 ± 12.0	0.16
CS	EV (mV)	1.5 ± 0.5	1.5 ± 0.3	0.14
	ED (ms)	30.7 ± 8.5	34.1 ± 6.3	0.45
	ΔEV (mV/s)	6.2 ± 2.9	6.3 ± 2.2	0.03
	ΔED (ms/ms)	29.2 ± 17.6	23.3 ± 11.2	0.40

Values are mean ± SD unless otherwise specified.

AF = atrial fibrillation; CS = coronary sinus; HRA = high right atrium; other abbreviations as in Table 1.

**Figure 8:**
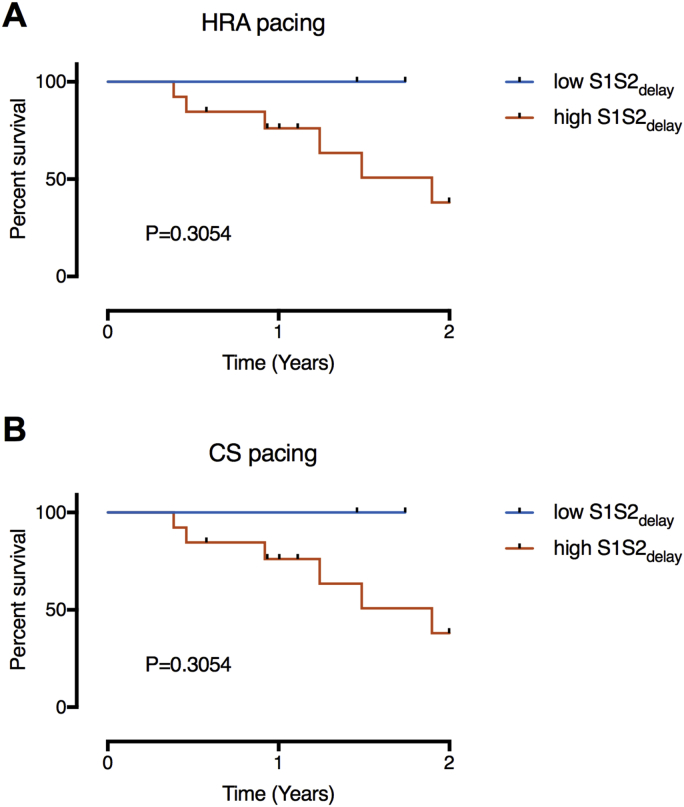
Kaplan-Meier Survival Analysis Survival curves are shown with patients dichotomized into groups based on the S1S2_delay_ populations identified in Figure 6 under HRA pacing **(A)** and CS pacing **(B)** conditions. Abbreviations as in Figures 1 and 2.

Kaplan-Meier survival curves representing time to arrhythmia recurrence for patients with low and high S1S2_delay_ (defined from the bimodal distributions illustrated in Figure 6) are shown in Figure 8. Although all arrhythmia recurrences were seen in patients with conduction delay occurring at long extrastimulus coupling intervals (long S1S2_delay_), the difference between the curves was nonsignificant by the log-rank test.

## Discussion

This study used detailed extrastimulus mapping to characterize coupling interval–dependent changes in LA conduction and electrogram morphology in patients with PAF. In a group of clinically similar patients with normal baseline LA voltage: 1) prolonged intra-atrial conduction is associated with increased activation dispersion, increased vulnerability to AF initiation, and recurrence of arrhythmia postablation; and 2) although a spectrum of electrogram morphological changes is also seen, significant variation with activation direction and extrastimulus coupling interval suggest they may be unreliable measures of AF substrate. Overall, these findings document significant electrophysiological variation between uniformly classified PAF patients.

Both a trigger and a substrate are implicated in the initiation and maintenance of AF (11). The concept of an AF substrate may explain variable clinical presentations whereby patients with more advanced remodeling are at greater risk of AF induction by spontaneous ectopy. Quantifying the AF substrate in practice is challenging, with clinical guidelines largely dependent on arrhythmia duration to guide management (1). Key components of the atrial substrate include altered refractoriness, electrical conduction, and atrial fibrosis.

### Altered refractoriness

In clinical studies of atrial refractoriness, ERP increases with both age and hypertension in the absence of AF 9, 12. In the presence of AF, ERP prolongs compared with controls (13), but as AF progresses from paroxysmal to persistent, measured ERP universally shortens (14). Hence, although shortening of refractoriness is associated with more severe forms of AF, prolongation of refractoriness may prevent AF in at-risk disease states. The data presented here show significant variation in ERP between cases. In agreement with these findings, previous work has shown that shortening of refractoriness, but not dispersion of refractoriness, is greater in chronic AF compared with PAF (15). Our study extends these findings to show that even within this group of uniformly classified PAF patients, significant intercase differences in refractoriness are identifiable. A spectrum of AF-related electrical remodeling may therefore be present in these patients.

### Electrical conduction

Previous in vitro work shows that discontinuous conduction, at a cellular level, may explain the occurrence of activation direction–dependent fractionation of extracellular potentials (16). The data presented here indicate that varying degrees of discontinuous conduction appear to occur in vivo in PAF patients: on a point-by-point basis, 3 patterns of local electrogram complexity exist: 1) absence of fractionation; 2) fractionation dependent on incident wave direction; and 3) fractionation independent of incident wave direction. These observations are in agreement with prior work demonstrating a lack of relationship between electrogram fractionation in sinus rhythm compared with CS pacing at a single cycle length (17). Notably, however, the occurrence of these fractionation patterns is dependent on the extra-stimulus coupling interval, with shorter coupling intervals required to reveal conduction discontinuities in some patients which are present much earlier in other patients. Quantification of the change in electrogram morphology (ΔED and ΔEV) with extrastimulus coupling interval confirmed responses that were markedly dependent on activation direction at some, but not all, recording sites. Such dependence of electrogram morphology on activation direction is consistent with the presence of more advanced remodeling at these recording sites (18). A number of possible mechanisms may underlie the occurrence of fractionated signals, with previous work indicating that EV may distinguish between fractionation occurring because of atrial fibrosis and fractionation occurring because of parasympathetic innervation (19). The present study extends these findings, suggesting that activation rate dependence of electrogram fractionation could be a useful tool in stressing atrial conduction and revealing underlying sites of conduction disturbance. The relationship between these fractionation patterns and underlying physiological/pathological mechanisms should be addressed in future studies.

Intra-atrial conduction delay, recorded at a single activation rate in the right atrium, has been shown to increase in patients with AF recurrence (20). In the present study, the parameter S1S2_delay_ was defined as the shortest S1S2 coupling interval conducting without delay to the atria and identifies the activation rate at which delay of the A2 electrogram becomes measureable. Possible contributors to the timing of S1S2_delay_ include delay close to the pacing site (related to the relative refractory period of the stimulated tissue) as well as conduction delay within the LA. Although prior studies have identified significant pacing site latency (21) as a cause of intra-atrial conduction delay, in the cases studied here pacing site latency (defined as conduction time within the CS) was insufficient to explain all the conduction delay seen. S1S2_delay_ was uniform throughout the LA, closely correlated under HRA and CS pacing conditions and bimodally distributed throughout the population studied.

To quantify conduction within the atria, the spread of activation times along the LA conducting path length at the full range of extrastimulus coupling intervals was determined. ADT was defined as the greatest increase in point-by-point local activation time as S1S2 was reduced. Sites close to earliest LA activation showed less dispersion of activation as coupling interval was reduced than sites distant from earliest LA activation. Cases that were AF-inducible at electrophysiology study showed greater activation rate dependent activation dispersion than cases that were not inducible. This later observation is consistent with prior work indicating that the absence of conduction velocity restitution is protective against fibrillatory conduction or AF initiation (22). S1S2_delay_ was positively correlated with ADT, which is explained by patients with higher S1S2_delay_ having a smaller range of S1S2 coupling intervals that conduct without delay throughout the left atrium. Although measurement of ADT requires panoramic mapping of the entire atrium, S1S2_delay_ can be measured at a single LA site and may therefore provide a reliable and clinically applicable measure of the response of intra-atrial conduction to changes in coupling interval.

### Atrial fibrosis

Electrogram voltage, recorded by contact mapping, correlates with increased atrial fibrosis in simulation studies (23), histological studies (24), and cardiac magnetic resonance imaging studies (25). In line with the concept of a fibrotic atrial cardiomyopathy, sinus rhythm atrial voltages are reduced in patients with AF (3), patients with increasing severity of AF (2), and patients with risk factors for AF, including age (12) and hypertension (9). However, other studies have failed to identify any difference in global and regional LA voltages between patients with and without AF (4).

### Study limitations

The data presented here show a spectrum of mean LA voltages both between patients and between LA regions. Previous studies found higher voltages in the posterior wall of the left atrium (26) or lower voltage in the posterior wall and septum (3). We again showed a regional variation (higher in the floor, posterior, and roof regions) in voltage, but this variation was only evident during HRA pacing, not during CS pacing. Such regional dispersion of LA voltage could be explained by preferential fibrosis of LA regions consequent on the occurrence of AF. However, although histological and imaging studies have identified regional variation in fibrotic remodeling in the left atrium — for example, showing greatest fibrosis in the posterior wall (27) — bipole voltage is also affected by a number of other factors including orientation of the recording bipolar to the activation wave, rhythm during mapping, and activation direction–dependent wave collision 28, 29. Overall, these observations suggest that bipolar voltage mapping alone may be insufficient to define the electrical substrate for AF and may therefore be an imperfect target for ablation strategies beyond pulmonary vein isolation alone.

## Conclusions

Incremental atrial pacing reveals significant electrophysiological variation between patients uniformly classified with PAF. Variation in conduction could be used during electrophysiological interventions to refine substrate-targeted ablation strategies and the ability to quantify such variation could help to stratify between ablation strategies.

Perspectives **COMPETENCY IN MEDICAL KNOWLEDGE:** Although AF classifications are based on arrhythmia duration, the clinical course and treatment response vary between patients within these groups. Electrophysiological mechanisms responsible for this variability are incompletely described. This study characterized direction-dependent and coupling interval-dependent changes in left atrial conduction in paroxysmal AF patients. A range of activation patterns that correlated with AF vulnerability and arrhythmia recurrence was identified. These findings illustrate that 1) even in apparently healthy atria as defined by voltage mapping, variability in left atrial activation (manifest as increased activation dispersion) may occur and 2) this variability may be responsible for the clinical spectrum of arrhythmia occurrence/recurrence seen in these patients.

**TRANSLATIONAL OUTLOOK:** Using currently-available techniques the identification of the mechanism of AF in humans is challenging. Instead, an approach identifying the electrophysiological substrate supporting AF could improve selection for ablation procedures. This work showed that extra stimulus testing (which could be thought of as an electrical stress test) can reveal atrial conduction abnormalities that are not evident during baseline atrial pacing. Quantification of activation dispersion using these techniques could therefore provide a new method for characterizing the atrial electrical phenotype in AF. Future work should establish methods to quantify rate-dependent activation dispersion using non-invasive tools such as electrocardiographic imaging.
