# Supplementary material for: Intra-Atrial Conduction Delay Revealed by Multisite Incremental Atrial Pacing is an Independent Marker of Remodeling in Human Atrial Fibrillation
Source: JACC Clin Electrophysiol. 2017 Sep;3(9):1006–17. doi: 10.1016/j.jacep.2017.02.012 (PMC5612260; doi:10.1016/j.jacep.2017.02.012)
Supplement: Online Figures 1–5 [file mmc1.docx]

Online Figure 1. Custom equipment for delivering the pacing protocol **A** – System diagram showing the computer, interface and standard clinical setup. **B** – Computer interface with optical isolator. **C** – Lab setup.


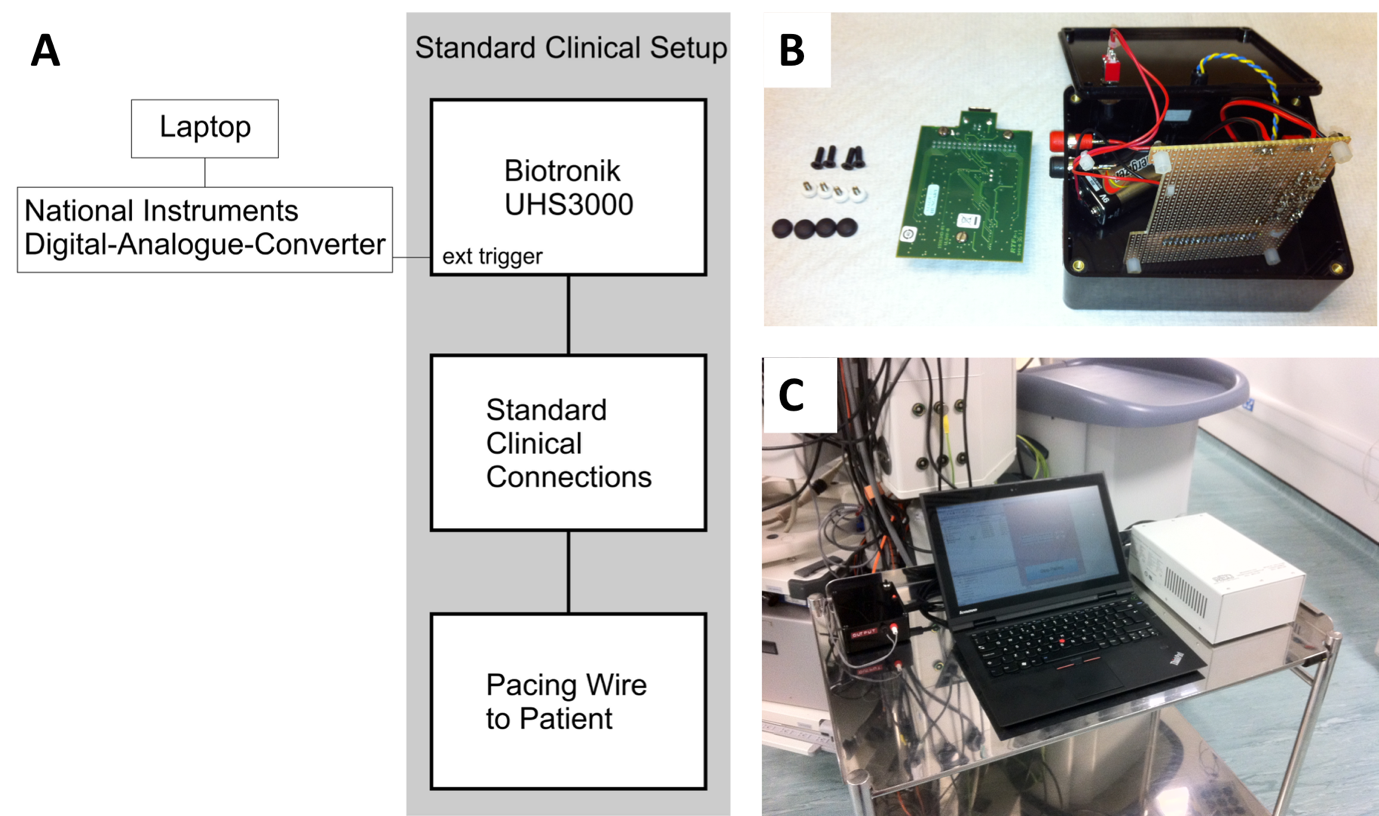


Online Figure 2. Optical isolator circuit diagram


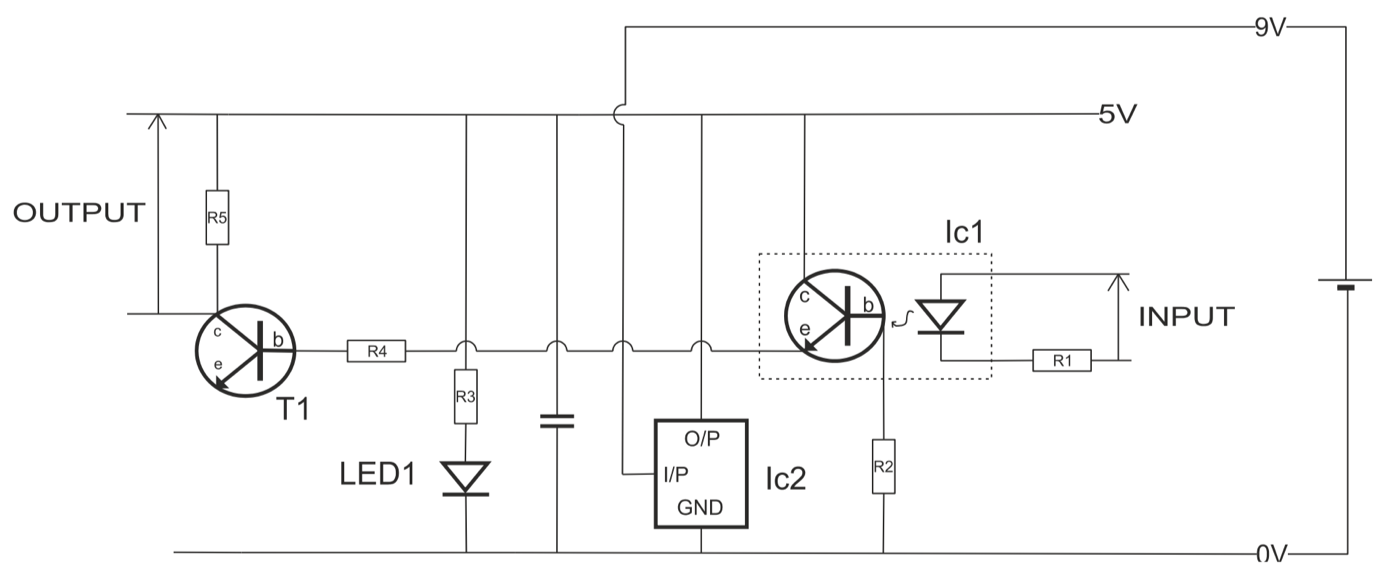


Online Figure 3. Path length computations Conducting path lengths were computed as geodesic distances (Mitchell et al. 1987) across the surface of the LA from earliest activation to recording site. The implementation of the exact geodesic algorithm was performed in Matlab using the library provided by D. Kirsanov (Kirsanov 2008).


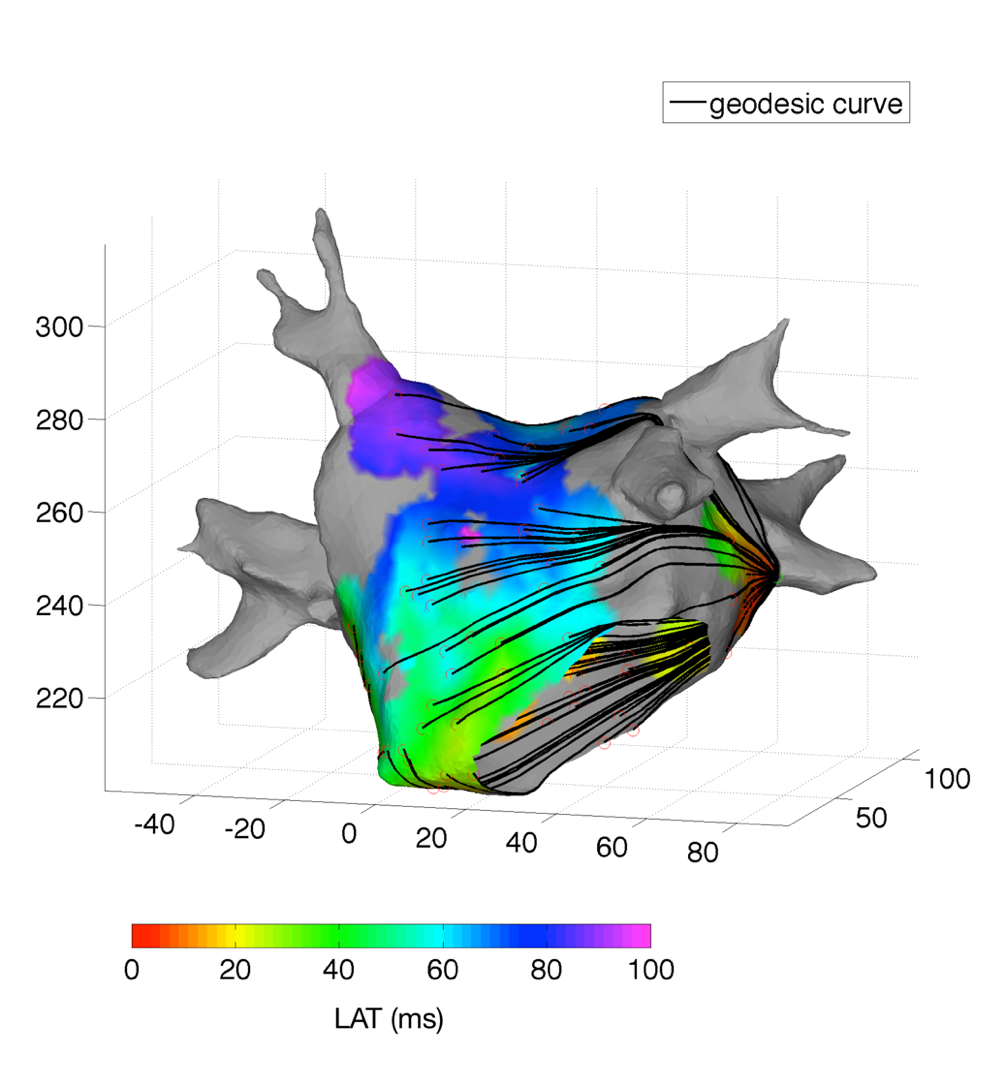


Online Figure 4. Electrogram Processing. **Left panel** – A complete drive train for a single site is shown with drive train electrograms (A1 egms, S1S2 coupling interval 480ms) shown in green with LA response electrograms (A2 egms, S1S2 coupling intervals decrementing from 343 to 200ms) shown in blue. Beat 79 (bottom) was labeled as ‘non-capture’ (red) and beat 81 was therefore automatically rejected from analysis. **Right panel** – complete pacing train with electrograms shown in blue, filtered electrograms in red and noise thresholds in green.


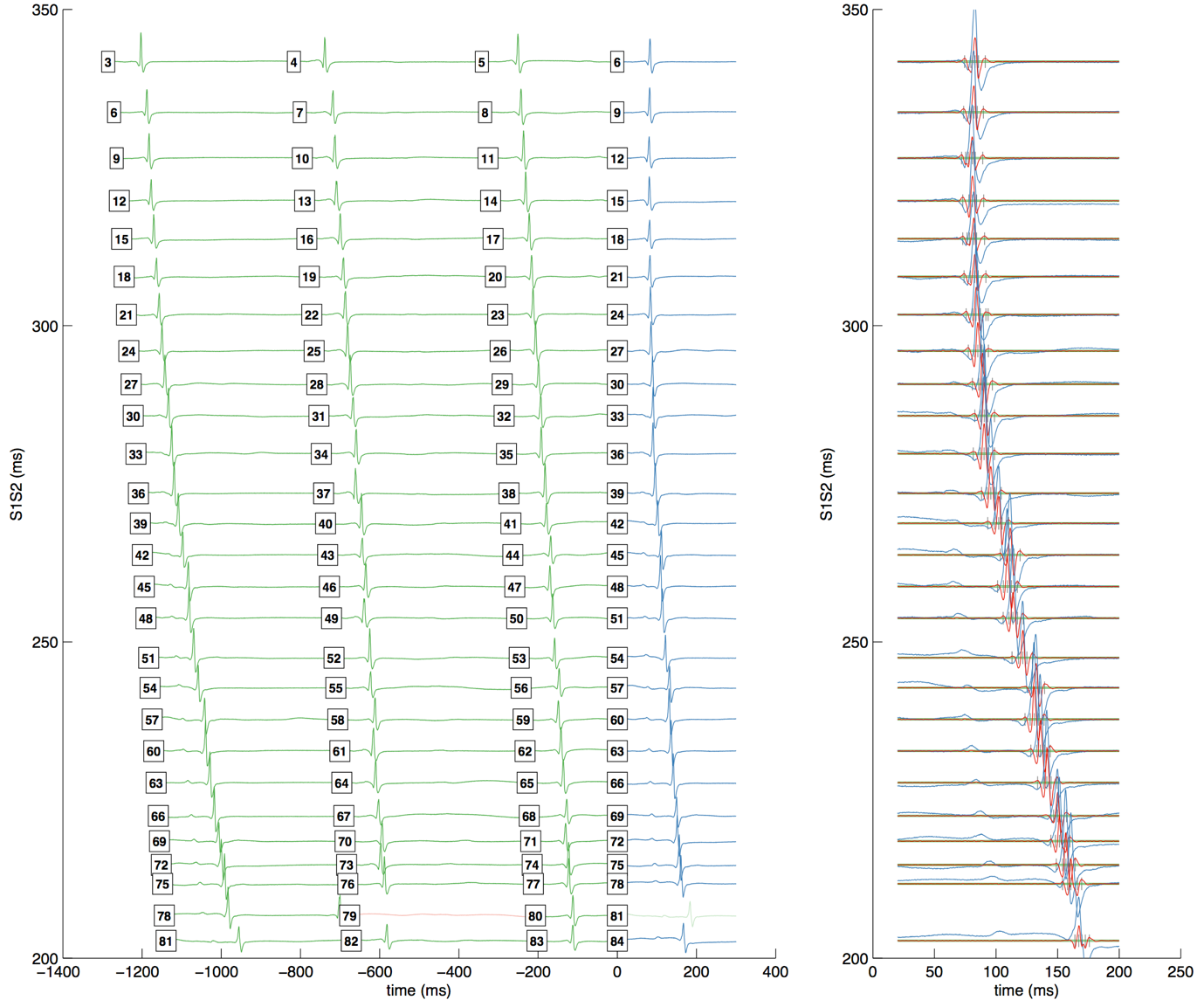


Online Figure 5. Definition of LA areas **A** – AP view. **B** – PA view. The LA was divided into 5 regions. *Posterior* - bounded by pulmonary veins; *Roof* - posterior to RSPV-RA line and anterior to RSPV-LSPV line; *Anterior* - from Roof to mitral annulus; *Floor* - from Posterior to mitral annulus; *Septum* - medial border of chamber between anterior and floor regions;


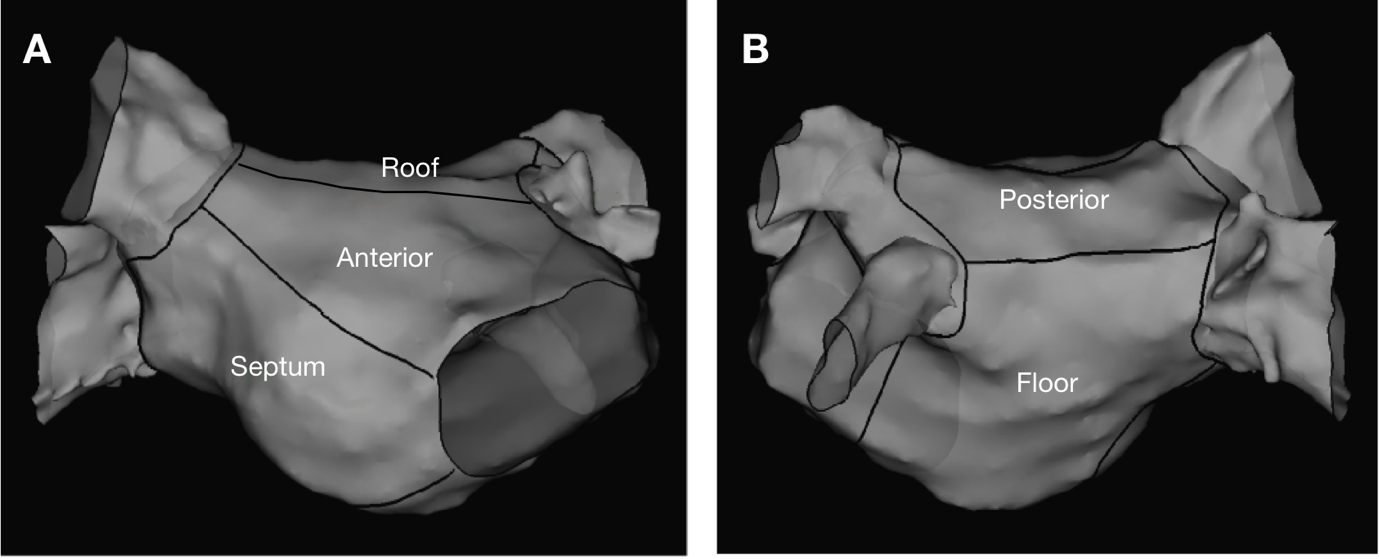


Kirsanov, D., 2008. Exact Geodesic for Triangular Meshes. Available at: http://uk.mathworks.com/matlabcentral/fileexchange/18168-exact-geodesic-for-triangular-meshes.

Mitchell, J., Mount, D. & Papadimitriou, C., 1987. The discrete geodesic problem. *SIAM Journal on Computing*, 16(4), pp.647–668.
